# A new species of *Tometes* Valenciennes 1850 (Characiformes: Serrasalmidae) from Tocantins-Araguaia River Basin based on integrative analysis of molecular and morphological data

**DOI:** 10.1371/journal.pone.0170053

**Published:** 2017-04-19

**Authors:** Marcelo C. Andrade, Valéria N. Machado, Michel Jégu, Izeni P. Farias, Tommaso Giarrizzo

**Affiliations:** 1 Universidade Federal do Pará, Cidade Universitária Professor José Silveira Netto, Instituto de Ciências Biológicas, Programa de Pós-Graduação em Ecologia Aquática e Pesca, Laboratório de Biologia Pesqueira e Manejo dos Recursos Aquáticos, Belém, Pará, Brazil; 2 Universidade Federal do Amazonas, Instituto de Ciências Biológicas, Programa de Pós-Graduação em Biodiversidade e Biotecnologia da Amazônia Legal, Rede Bionorte, Laboratório de Evolução e Genética Animal, Manaus, Amazonas, Brazil; 3 Institut de Recherche pour le Développement, Biologie des Organismes et Ecosystèmes Aquatiques, UMR BOREA, Laboratoire d´Icthyologie, Muséum national d’Histoire naturelle, MNHN, CP26, 43 rue Cuvier, Paris, France; Institute of Botany, CHINA

## Abstract

A new large serrasalmid species of *Tometes* is described from the Tocantins-Araguaia River Basin. *Tometes siderocarajensis* **sp. nov.** is currently found in the rapids of the Itacaiúnas River Basin, and formerly inhabited the lower Tocantins River. The new species can be distinguished from all congeners, except from *T*. *ancylorhynchus*, by the presence of lateral space between 1st and 2nd premaxillary teeth, and by the absence of lateral cusps in these two teeth. However, *T*. *siderocarajensis* **sp. nov.** can be differentiated from syntopic congener *T*. *ancylorhynchus* by an entirely black with mottled red body in live specimens, densely pigmented pelvic fins with a high concentration of dark chromatophores, and the presence of 39 to 41 rows of circumpeduncular scales (*vs*. silvery body coloration with slightly reddish overtones on middle flank during breeding period in live specimens, hyaline to slightly pale coloration on distalmost region of pelvic fins, and 30 to 36 rows of circumpeduncular scales). Additionally, molecular sequence shows that *T*. *siderocarajensis* **sp. nov.** is reciprocally monophyletic, and diagnosable from all congeners by having two autapomorphic molecular characters in the mitochondrial gene COI. The phylogenetic reconstruction still show that *T*. *siderocarajensis* **sp. nov.** is closely related to *T*. *trilobatus*. This is the first molecular study using an integrative taxonomic approach based on morphological and molecular sequence data for all described species of *Tometes*. These findings increase the number of formally described species of *Tometes* to seven. A key to the *Tometes* species is provided.

## Introduction

Serrasalmidae is a Cis‒Andean fish family that comprises more than 80 species, of which one is a fossil [1–3]. The serrasalmid species are easily recognized by having a very deep body (sometimes like a disk), often silvery in color, and scales modified into spines that generally form a ventral serrae [4–6]. The family is phylogenetically divided into three major clades, corroborated by both morphological and molecular studies: one composed by large herbivores of the genera *Colossoma*, *Piaractus*, and *Mylossoma*; another collectively referred as “*Myleus*” which is comprised mostly by herbivorous fishes from rapids; and a third composed of the famous ‘piranhas’ including the aquarium trade fishes ‘silver dollars’ of the genus *Metynnis* [3,7,8].

*Myleus* clade, *sensu* morphological phylogeny [7], is formed by the genera *Myleus*, *Mylesinus*, *Ossubtus*, *Tometes*, and, according to molecular phylogenies [8,9], some species of the genus *Myloplus*. With the exception of the latter genus, *Myleus* clade is comprised of strictly rheophilic species and is characterized by having incisiform teeth on the jaws, two premaxillary rows of teeth that maintain inner contact, and prepelvic serra composed of thin spines not forming an abdominal keel (absent in *Ossubtus xinguense*) [10,11]. The genus *Tometes* was taxonomically hidden for many years [12], and was hence mistaken with other serrasalmid genera, most notably *Utiaritichthys*, a genus that some authors consider rare and poorly-known (e.g. Gosline [13], Géry [4,14], and Goulding [15]). Recently, most of these assignments were reported as misidentifications of *Tometes* because both genera show diminute prepelvic spines. However, *Utiaritichthys* is strictly distinguished from *Tometes* by having molariform teeth with two premaxillary teeth rows interspaced by a gap *versus* incisiform teeth with two premaxillary teeth rows lacking inner gap [11]. *Tometes* contains six valid species distributed in South America along drainages of Brazilian and Guiana Shields [11].

During analyses of specimens from Mosaic of Conservation Units (MCU) of the Serra dos Carajás, Itacaiúnas River, a left-bank tributary of lower Tocantins River Basin, as well as some specimens collected in lower Tocantins River (prior to the flooding from the Tucuruí Hydroelectric reservoir) a new species of *Tometes* was discovered and described herein.

## Material and methods

### Ethics statement

Statement from an ethics committee was not necessary, once the analysis did not involve endangered or protected species. Except from the specimens deposited under institutions, which tissues were extracted from specimens collected with appropriate permissions under authorizations numbers 11325–1 and 38263–1 issued by ICMBio (Chico Mendes Institute for Biodiversity Conservation), and also 045/2008-2011 issued by IBAMA (Brazilian Institute of Environment and Renewable Natural Resources).

### Nomenclatural acts

The electronic edition of this article conforms to the requirements of the amended International Code of Zoological Nomenclature, and hence the new names contained herein are available under that Code from the electronic edition of this article. This published work and the nomenclatural acts it contains have been registered in ZooBank, the online registration system for the ICZN. The ZooBank LSIDs (Life Science Identifiers) can be resolved and the associated information viewed through any standard web browser by appending the LSID to the prefix “http://zoobank.org/”. The LSID for this publication is: urn:lsid:zoobank.org:pub: 69CDF38A-05CD-4351-8791-91889B741DE2. The electronic edition of this work was published in a journal with an ISSN, and has been archived and is available from the following digital repositories: PubMed Central, LOCKSS.

### Morphological analyses

Counts and measurements follow Jégu et al. [16,17] and were taken whenever possible on left side of specimens. Counts are given in description as the range of counts followed by the value observed in holotype in parentheses. Standard length (SL) is expressed in millimeters; subunits of body are showed as percentage of SL, and the subunits of the head as percentage of head length (HL). Osteological description, vertebral and supraneurals analysis were obtained from two dry skeletons (labeled as “skel.” in material examined). Osteological terminology follows Weitzman [18] with modifications of Mattox et al. [19]. Vertebral counts include the Weberian apparatus as four elements, and the compound caudal centrum (PU1+U1) is counted as one element. Institutional abbreviations are as follows: CAS (California Academy of Sciences, San Francisco); CTGA (Laboratório de Evolução e Genética Animal, Universidade Federal do Amazonas, Manaus); GEA (Laboratório de Ictiologia do Grupo de Ecologia Aquática, Universidade Federal do Pará, Belém); IEPA (Instituto de Ensino Profissional da Amazônia, Macapá); INPA (Instituto Nacional de Pesquisas da Amazônia, Manaus); INRA (French National Institute for Agricultural Research, Paris); MNHN (Muséum national d’Histoire naturelle, Paris); MPEG (Museu Paraense Emilio Goeldi, Belém); MZUSP (Museu de Zoologia da Universidade de São Paulo, São Paulo); TAMU (Texas Agricultural & Mechanical University, College Station); and ZUEC (Museu de Zoologia da Universidade Estadual de Campinas ‘Adão José Cardoso’, Campinas).

### Molecular analyses

The molecular analyses were made using 28 specimens of the six valid *Tometes* plus the new species proposed herein, from six large tributaries of the Amazon Basin in Brazil: as Jari, Negro, Tapajós, Tocantins-Araguaia, Trombetas and Xingu river basins; and also from Maroni River, a costal drainage between French Guiana and Surinam. Tissues were preserved in 95% ethanol for DNA extraction and deposited at CTGA, with vouchers deposited at GEA, IEPA, INPA and MPEG. The sequences obtained in this study are deposited in GenBank under the following accession numbers KX868671 to KX868698.

Total DNA was isolated from approximately 50mg of tissue using standard phenol-chloroform extraction methods [20]. A fragment of approximately 750 bp of the mitochondrial control region was amplified using the primers LPROF (5' AACYCCCRCCCCTAACYCCCAAAG 3') and DLOsteri R1 (3' GTAAAACGACGGCCAGTCCTGGTTTH 5'). About 670 bp mitochondrial region of the cytochrome C oxidase subunit I (COI) was amplified using the M13-tailed primer cocktails FishF2_FishR2 and VF2_VR1d [21]. The 15 μl polymerase chain reaction (PCR) mix included 1.2 μL of 10 mM dNTPs (2.5 mM each DNTP), 1.5 μL 10X buffer (75mM Tris HCL, 50 mM KCL, 20 mM (NH_4_)_2_SO_4_), 1.2 μL 25 mM MgCl_2_, 1.5 μL of primer cocktails (2 pM each) for COI and 1.5 μL of each primer for DLoop, 0.5 μL of Taq DNA polymerase, 1 μL of template DNA and 6.6 μL ddH2O. PCR conditions were as follows: 94°C (30 sec), 35 cycles of 94°C (30 sec), 50°C (35 sec), 72°C (1:30 min), followed by 72°C (5 min). Amplicons obtained were submitted for purification and the sequencing was performed in an automatic ABI 3500 sequencer (Applied Biosystems).

The forward and reverse COI and control region chromatograms were assembled into contigs using Geneious 7.0.6 [22] and edited manually. The alignment was then checked manually for insertions, deletions or stop codons for COI sequences using translated amino acids. Genetic distances (uncorrected *p*-distances, recommended by Collins et al. [23]) were calculated using the Ape 3.5 package [24] in R version 3.3.2 [25], with the pairwise deletion option set to “true”. We used a cutoff of 2.0% for genetic distance as a threshold value adopted for the COI-based identification systems [26] in most Neotropical freshwater fish fauna [27]. We further visualized the divergence of these taxa using a Neighbor Joining (NJ) tree, which is the standard method of phylogenetic inference in DNA barcoding studies [26]. To demonstrate that the new species is divergent and diagnosable from all other nominal species of *Tometes*, assuming the Phylogenetic Species Concept [28], we used the R package SPIDER [29] to extract diagnostic molecular characters in the COI and control region sequences. Additionally, we concatenated the two genes and analyze the phylogenetic relationships among the species using a maximum-likelihood (ML) in the Ape 3.5 package [24]; the nucleotide substitution model (GTR+G) was selected using Phangorn with the AICc criterion, and node support was evaluated using 1,000 bootstrap replicates. In the ML analysis we included *Myloplus schomburgkii* and *Myloplus rubripinnis* as outgroups. Sequence alignment is available at https://github.com/legalLab/datasets.

## Results

### *Tometes siderocarajensis* sp. nov.

urn:lsid:zoobank.org:act:2DF2F54C-E255-40A6-811F-06E0BABD5543

*Tometes* sp. Tocantins: ‒Andrade et al. [30]: page 4 in figure 2B (premaxillary in labial view, and comparative material utilized).

**Fig 1:**
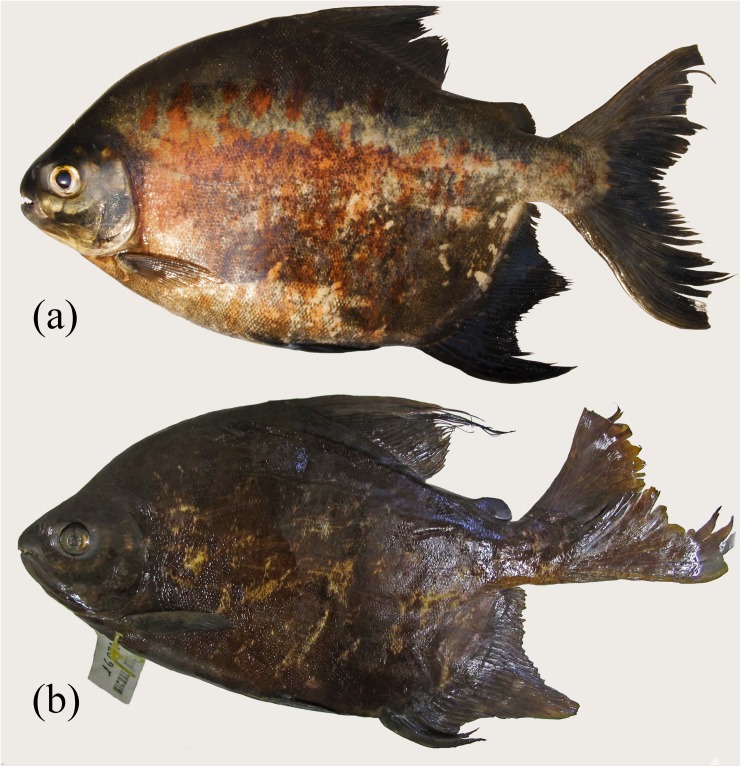
*Tometes siderocarajensis* sp. nov. from Tocantins-Araguaia River Basin, (a) MPEG 33922, holotype photographed alive, male, 338.0 mm SL; (b) MZUSP 117052, paratype, preserved specimen, male, 341.3 mm SL.

**Fig 2:**
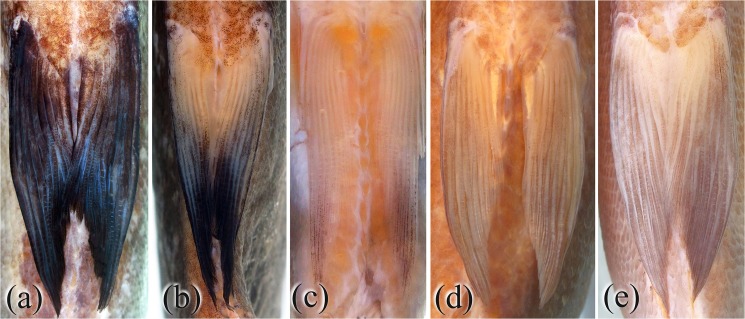
Ventral view of pelvic fins: *Tometes siderocarajensis* sp. nov. (a) MPEG 33925, paratype, male, 332.0 mm SL, and (b) GEA 1944, paratype, female, 200.3 mm SL. *Tometes ancylorhynchus*, (c) GEA 1955, female, 158.5 mm SL. *Tometes lebaili*, (d) MNHN 1993‒3452, female, 251.0 mm SL. *Tometes trilobatus*, (e) MNHN 1998–0099, female, 248.0 mm SL.

**Fig 3:**
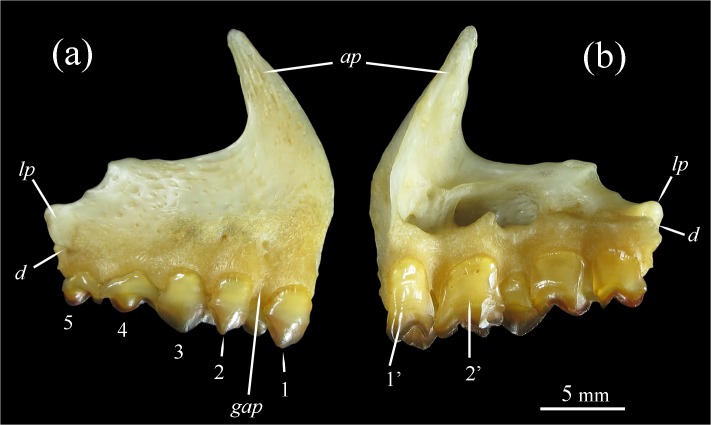
Premaxilla of the *Tometes siderocarajensis* sp. nov., GEA 1945, female, 280.0 mm SL, (a) lateral view, (b) internal view. 1‒5: Labial premaxillary row; 1’‒2’: Lingual premaxillary row; *ap*: Ascending process of premaxilla; *lp*: Lateral process of premaxilla; *d*: Dimple of articulation with anterodorsal portion of maxillary; gap: lateral space between 1 and 2.

**Fig 4:**
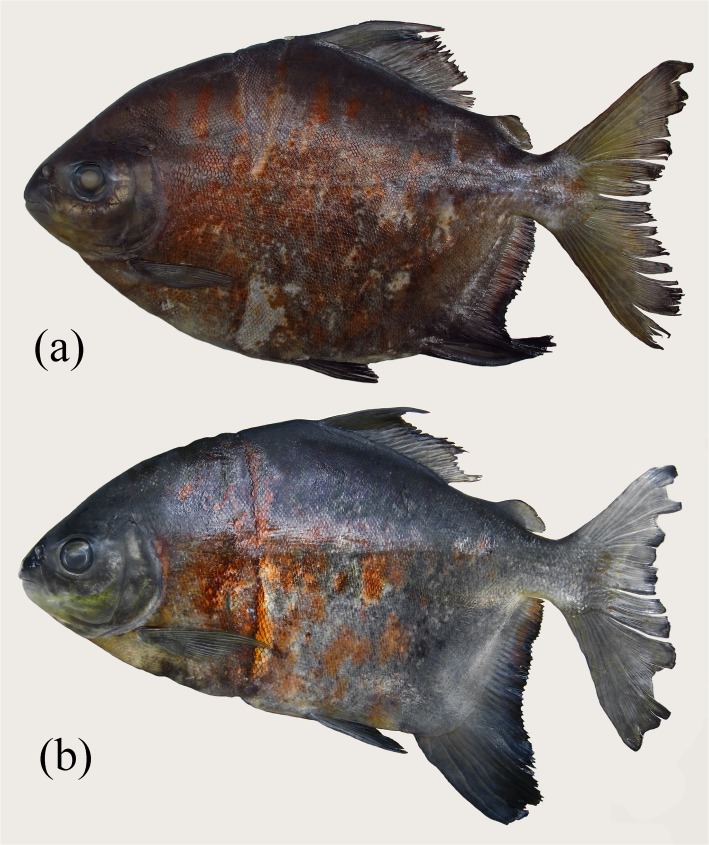
*Tometes siderocarajensis*, paratypes, (a) INPA 52811, female, 227.3 mm SL, (b) ZUEC 12598, female, 328.0 mm SL. Preserved specimens.

Fig 1A, Fig 1B, Fig 2A, Fig 2B, Fig 3, Fig 4A and Fig 4B

#### Holotype

MPEG 33922 (1, 338.0 mm SL), Brazil, Pará, Parauapebas, Serra dos Carajás, Itacaiúnas River, nearby Paulo Fontelles Road, Caldeirão, 5°52'38.2"S 50° 29'27.8"W, Tocantins-Araguaia River Basin, 7 Sep 2010, D. Bastos & A. Jesus.

#### Paratypes

All from Brazil, Pará, Tocantins-Araguaia River Basin. GEA 1990 (1 skel., 340.0 mm SL), 3 Jul 2010, M. Andrade & A. Jesus, and MPEG 33925 (1, 332.0 mm SL), Oct 2008, D. Bastos & A. Jesus; same locality of holotype. GEA 1936 (1, 176.3 mm SL), same locality of holotype, 4 Jul 2008, T. Giarrizzo & A. Jesus. GEA 1942 (1, 306.1 mm SL), Parauapebas, Serra dos Carajás, Itacaiúnas River, downstream igarapé Salobo, Vira Mundo, 5°50'32.2"S 50°26'39"W, 20 Mar 2010, M. Andrade & A. Jesus. GEA 1944 (1, 200.3 mm SL), Parauapebas, Serra dos Carajás, Itacaiúnas River, upstream igarapé Cinzento, 5°53'07.9"S 50°31'54.6"W, 2 Jul 2008, T. Giarrizzo & A. Jesus. GEA 1945 (1 skel., 280.0 mm SL, Serra dos Carajás, Itacaiúnas River, Casa PAE, ICMBio, 5°55'44.7"S 50°43'2.6"W, Jun 2011, D. Ribeiro. ZUEC 12598 (2, 328.0‒352.0 mm SL), same data as GEA 1945. INPA 52481 (7, 84.9‒278.0 mm SL), Tocantins River, Itupiranga, Nov 1980, M. Jégu. INPA 52811 (1, 277.3 mm SL), Parauapebas, Serra dos Carajás, Itacaiúnas River, downstream igarapé Salobo, Vira Mundo, 5°50'32.2"S 50°26'39"W, Jun 2011, M. Andrade & A. Jesus. MPEG 33923 (2, 281.3‒335.9 mm SL), Parauapebas, Serra dos Carajás, Itacaiúnas River, downstream igarapé Salobo, Vira Mundo, 5°50'32.2"S 50°26'39"W, 17 Dec 2009, M. Andrade & A. Jesus. MPEG 33924 (1, 287.5 mm SL), Ourilândia do Norte, Serra Onça‒Puma, Cateté River, 6°34'1.2"S 51°01'57.1"W, 6 Jun 2011, A. Jesus. MZUSP 117052 (11, 248.5‒341.3 mm SL), Parauapebas, Serra dos Carajás, Itacaiúnas River, Caldeirão, Nov 1983, M. Goulding.

#### Diagnosis

*Tometes siderocarajensis* **sp. nov.** is distinguished from all congeners by dense pigmentation on the distalmost portion of the pelvic-fin rays or the entirety of the fin (Fig 2A, Fig 2B) [*vs*. pelvic fin hyaline or pale, or with few scattered dark chromatophores along distal portion of rays (Fig 2C, Fig 2E)]. Additionally, it is distinguished from all, except from *T*. *camunani* and *T*. *kranponhah*, by having more circumpeduncular scale rows (39‒41 *vs*. 38 or less), and from *T*. *kranponhah* and *T*. *trilobatus* by having 1st and 2nd labial premaxillary teeth laterally spaced (Fig 3A) (*vs*. 1st and 2nd labial premaxillary teeth with lateral contact). The new species is further distinguished from *T*. *trilobatus* by having more perforated lateral line scales (74–84 *vs*. 58–72) and from *T*. *makue* by having more spines on ventral keel (11–17 prepelvic spines and 26–33 total spines *vs*. 0–9 and 10–23, respectively). Finally, *Tometes siderocarajensis* **sp. nov.** can be distinguished from *T*. *lebaili* by having a terminal to gently upturned mouth and invariably 5 dentary teeth (*vs*. a markedly upturned mouth and 6–7 dentary teeth).

#### Description

Morphometric data presented in Table 1. Serrasalmid medium to large sized. Deep body, elongated to ovoid (Fig 1A, Fig 1B), laterally compressed. Greatest body depth at dorsal-fin origin. Snout tip slightly rounded. Dorsal profile of head straight to gently convex from vertical through nostrils to supraoccipital spine end. Dorsal-fin base straight to convex and interdorsal profile straight to gently concave. Ventral head and body profiles convex. Abdomen lacking ventral keel, thin prepelvic spines under skin. Prepelvic spines 11‒17 (17). Postpelvic spines 7‒12 (8), and paired spines around anus 5‒8 (8). Total spines 26‒33 (28). Anal-fin base straight to gently convex in females and strongly convex in males.

**Table 1:** Morphometric data for *Tometes siderocarajensis* sp. nov. Range of measurements includes the holotype (MPEG 33922). Hol, holotype; n, number of specimens; SD, standard deviation.

	Hol	n	Range	Mean±SD
Standard length	338.0	31	84.9‒352.0	280.3
	*Percentage of standard length*			
Body depth	55.3	29	53.9‒64.7	59.5±3.1
Head length	22.8	29	22.6‒30.7	24.7±1.5
Distance from snout to supraoccipital spine	27.6	29	27.1‒33.0	29.8±1.3
Predorsal length	53.7	29	53.3‒59.6	57.5±1.6
Dorsal-fin base length	28.1	29	27.1‒31.8	29.6±1.1
Interdorsal length	10.7	29	9.0‒13.3	11.4±0.9
Adipose-fin base length	5.4	29	5.0‒7.0	6.1±0.6
Caudal-peduncle depth	10.5	29	10.5‒12.1	11.2±0.5
Caudal peduncle width	3.4	29	2.6‒5.6	3.9±0.5
Prepectoral length	22.2	29	21.0‒25.7	23.4±1.0
Pectoral-fin length	19.5	29	19.2‒23.3	21.5±1.1
Pelvic-fin origin to anal-fin origin	19.6	29	18.8‒24.2	21.7±1.5
Pectoral-fin origin to pelvic-fin origin	30.7	29	30.1‒34.7	32.7±1.3
Prepelvic length	52.6	29	52.6‒58.6	55.6±1.5
Pelvic-fin length	15.7	29	15.1‒19.4	17.2±1.0
Preanal length	70.1	29	70.1‒79.4	75.4±2.5
Anal-fin base length	29.3	29	29.3‒34.2	31.6±1.3
Second anal-fin lobe length	15.2	12	13.1‒22.4	18.2±3.5
Dorsal-fin lobe length	23.4	24	20.5‒38.8	28.1±4.3
Dorsal-fin origin to anal-fin origin	59.0	29	58.8‒70.1	63.8±3.1
Dorsal-fin end to anal-fin origin	43.2	29	42.0‒52.3	47.1±2.4
Dorsal-fin end to anal-fin end	23.0	29	23.0‒28.1	25.5±1.2
	*Percentage of head length*			
Snout length	35.5	29	27.5‒39.8	34.6±3.3
Mouth length	27.1	29	16.2‒28.8	23.2±2.6
Mouth width	38.0	29	30.8‒41.1	36.8±2.7
Interorbital width	51.0	29	39.3‒53.6	49.7±3.1
Head width	66.2	29	48.2‒69.3	62.4±3.9
Third infraorbital width	12.5	29	8.5‒14.7	11.3±1.2
Fourth infraorbital width	13.4	29	11.8‒17.2	14.3±1.2
Eye vertical diameter	30.0	29	25.2‒39.1	30.9±3.3
Postorbital distance	31.0	29	23.7‒35.9	30.9±2.4

Mouth terminal to somewhat upturned, premaxillary slightly longer than dentary. Incisiform teeth. Premaxillary with 5 labial teeth and 2 lingual teeth (Fig 3A, Fig 3B). Labial premaxillary row abutting with lingual premaxillary row. First to 3rd teeth of labial premaxillary row high, without lateral cusps, and crows in ventral view with subtle curve; 4th and 5th teeth, smaller, tricuspids, and crows in ventral view forming sigmoid shape. First two teeth of labial premaxillary row laterally spaced. Dentary with 5 teeth on main row, fitted between the two rows of premaxillary teeth, and pair of symphyseal teeth. Dentary elongated, thin anteroposteriorly, gently arched with five bony lamellae at symphysis. Maxillary edentulous.

Scales cycloid, irregular sized. Perforated lateral line scales from supracleithrum to hypural plate end 74‒84 (80), and total perforated lateral line scales 79‒90 (86). Horizontal scale rows between dorsal-fin origin and lateral line 45‒53 (50). Horizontal scale rows between lateral line and pelvic-fin insertion 42‒41 (46). Circumpeduncular scales rows 39‒41 (40).

Dorsal fin preceded by forward directed spine. Dorsal-fin rays ii‒iii (iii), 20‒22 (21). Distal margin of dorsal fin falcate with filaments in some cases (see under sexual dimorphism). Anal-fin rays iii-iv (iii), 31‒35 (34). Pectoral-fin rays i, 15‒17 (16). Pelvic-fin rays invariably i, 7. Adipose fin present, with oblique base, distal margin gently straight, sub-rectangular shaped. Caudal fin forked with similarly-sized lobes, almost reaching body depth when vertically stretched. Five to six supraneurals. Forty-two total vertebrae. Nine predorsal vertebrae, and 16 postdorsal vertebrae. Two vertebrae between vertical through last dorsal fin pterygiophore and first anal-fin pterygiophore. First branchial arch with 28‒29 gill rakers, 12‒13 on upper branch; one at cartilage between ceratobranchial and epibranchial, and 14‒16 on lower branch.

Neurocranium in lateral view as high as long, triangular, and with gently concavity at epiphyseal bar. Fontanells equally sized. Mesethmoid trapezoid, elongated forward with anterior process pointed and directed downward. Ethmoidal wings elongated forward, positioned on anterior half of mesethmoid. Wide olfactory fossae, and slender roof of mesethmoid.

#### Coloration in alcohol

Ground color brown darkish with black and red blotches scattered on flanks (Fig 1A, Fig 1B, Fig 4A, Fig 4B). Some specimens can present pale coloration due to fading from alcohol and light (Fig 1B). Dorsal portion of head and flanks darker than lower portion. Portion of pelvic fins and first rays of anal fin densely blackened by presence of chromatophores (distalmost portion of pelvic-fin rays densely pigmented, or whole fin completely dark colored). Distal margin of caudal and dorsal fins conspicuously dark colored. Pectoral fins hyaline, and adipose fin with distal margin slightly darkened.

#### Coloration in life

Overall color pattern brown with black and red blotches scattered on flanks. Dorsal profile of head, cheek gap, middle zone of opercle, and joint between operculum and subopercle with high concentration of dark chromatophores. Pectoral fins uniformly light brown, while adipose and caudal fins darker pigmented, and pelvic and anal fins densely blackened.

#### Sexual dimorphism

*Tometes siderocarajensis* displays secondary sexual features. The 17 mature males examined have an additional lobe formed by the middle branched anal-fin rays (Fig 1A, Fig 1B). Additional lobe centered on 14th‒17th (16th) branched rays (Fig 1A, Fig 1B). The females do not have this additional lobe and show a falcate anal-fin distal margin (Fig 4A, Fig 4B). First lobes of dorsal- and anal-fin rays variable in length between sexes (females with dorsal fin ranging from 20.5‒31.2% SL ± 3.5; and anal fin 24.4‒32.8% SL ± 2.6; and males with dorsal fin ranging 23.2‒30.1% SL ± 2.5; and anal fin ranging 20.2‒31.3% SL ± 3.6). Four of the 17 males, measuring 280 mm SL or more [including the holotype], exhibit stiff hooks laterally curved on the distalmost lepidotrichia of the anal-fin rays, and six males, the largest measuring 300 mm SL, have dorsal fin with very thin elongations (Fig 1B), ranging 4.6‒15.8% SL.

#### Molecular results

The control region sequences length was approximately 730 bp, including 162 variable sites, of which 132 were parsimony informative. The COI sequence length was about 580 bp with no observed insertions, deletions or stop codons. Out of 103 variable sites, 83 were parsimony informative. All species had maximum intra-specific divergence values below 1.0%, except *T*. *makue* and *T*. *trilobatus*, each of which was represented by a single sample. The uncorrected *p*-distances divergence ranged from 0.0% to 0.9% (mean 0.2%) for intra-specific comparisons and from 1.6% to 9.1% (mean 5.8%) for congeneric comparisons. Using a cutoff of 2.0% for delimiting species, a pair of species (i.e. *T*. *siderocarajensis* and *T*. *trilobatus*) showed interspecific values below this limit and could not be discriminated using this threshold alone. The ND characters obtained for COI sequences (S1 Table) is also used as a complementary analysis [31] to reinforce the utility of the DNA barcoding technique to identify species for those with low uncorrected *p*-distances divergence values (< 2.0%), such as *T*. *siderocarajensis* and *T*. *trilobatus*, which showed two exclusive NDs each, in 83 informative sites (S1 Table). However, no exclusive NDs were observed for the control region sequences to differentiate this pair of species.

**Fig 5:**
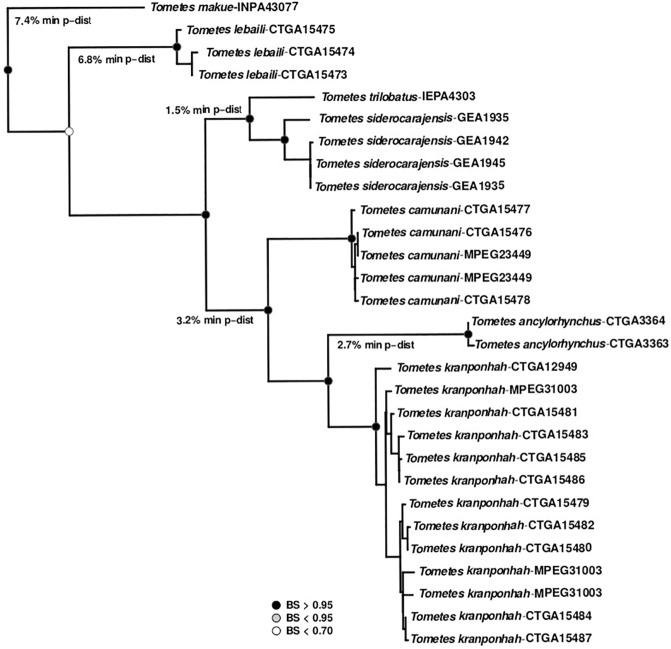
Neighbor-joining (NJ) tree of 28 mitochondrial cytochrome c oxidase subunit I gene sequences from seven *Tometes* species using uncorrected *p*-distance, showing the correct discrimination by distance genetic approach of the all species. Bootstrap values based on 1000 replicates are indicated at the branches.

The neighbor-joining (NJ) topology showed that all species in this study are reciprocally monophyletic with high bootstrap values (Fig 5). The following valid *Tometes* species were readily distinguishable using the DNA barcoding approach: *T*. *makue* was recovered as sister group of *T*. *lebaili*. In turn, this clade was recovered as sister group of a more inclusive clade comprised of two other groups, the first of which includes *T*. *trilobatus* and *T*. *siderocarajensis*, and the second of which includes *T*. *camunani*, *T*. *ancylorhynchus*, and *T*. *kranponhah*. *Tometes makue* and *T*. *ancylorhynchus* presented the highest interspecific distances values (9.1%). Among congeners, *T*. *trilobatus* was more closely related to *T*. *siderocarajensis* showing the lowest interspecific distance (1.6%).

**Fig 6:**
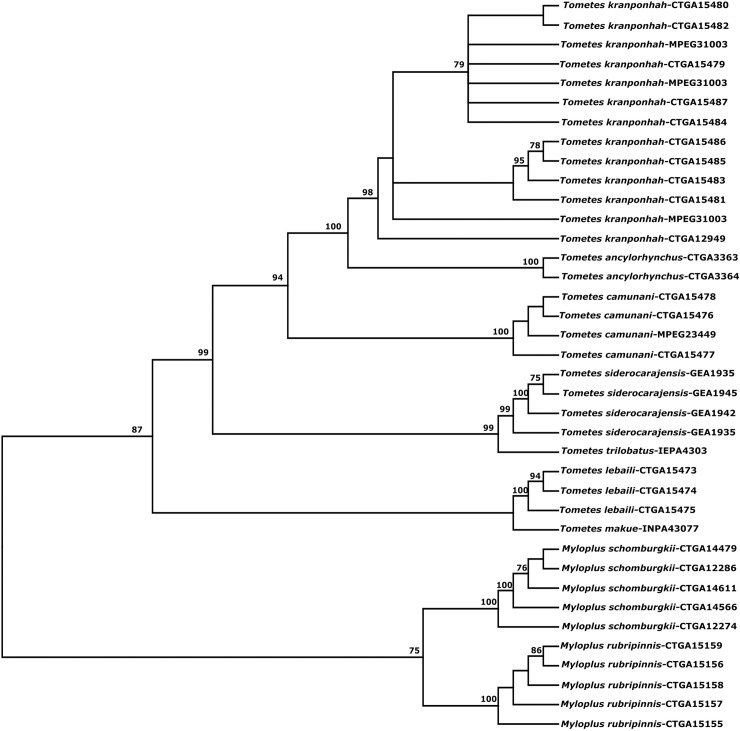
Maximum Likelihood tree inferred in RAxML using the concatenated data matrix of two mitochondrial genes (COI and Control Region), showing phylogenetic relationships within the genus *Tometes* genus. The numbers above branches are bootstrap probabilities > 50%. *Myloplus schomburgkii* and *Myloplus rubripinnis* were used as outgroups.

Based on the concatenated sequences of the two genes, ML tree (Fig 6) shows that all *Tometes* species are monophyletic, pending confirmation of *T*. *trilobatus* and *T*. *makue* since only one specimen was available for analysis. Relationships among species are well supported except for the sister taxon relationship of *T*. *lebailli* and *T*. *makue*.

#### Etymology

The epithet *siderocarajensis* alludes to the locality ‘Serra and Carajás’, which is the largest high-grade iron deposit in the world. From the Greek‒Latin *sidero* means ‘iron’, and *carajensis* in allusion to the type locality. A toponymic adjective.

#### Geographic distribution

**Fig 7:**
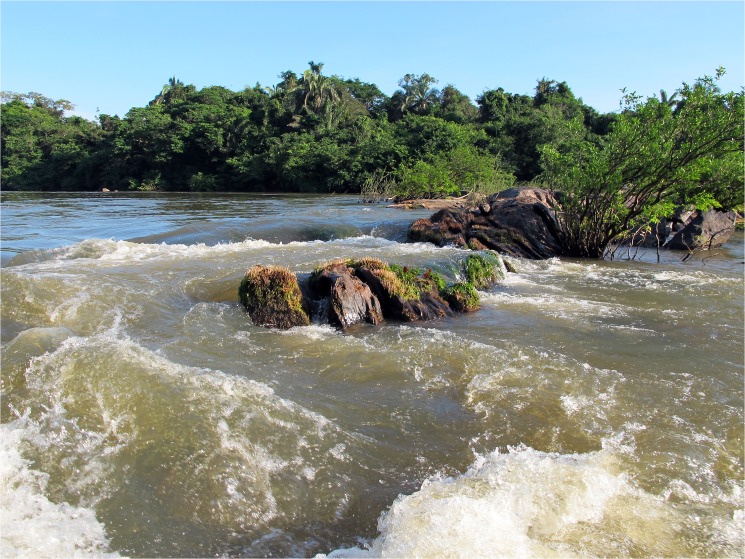
Itacaiúnas River, Pará State, at Mosaic of Conservation Units of Serra dos Carajás, type locality of *Tometes siderocarajensis*.

**Fig 8:**
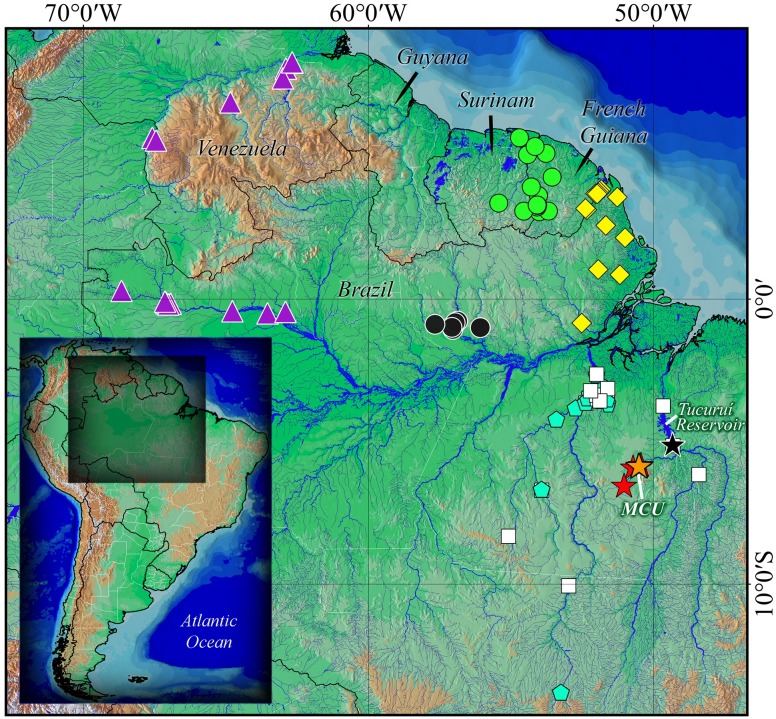
Distribution map of *Tometes* species. *Tometes ancylorhynchus* (white squares), *T*. *kranponhah* (blue pentagon), *T*. *makue* (purple triangle), *T*. *camunani* (black circle), *T*. *lebaili* (green circle), and *T*. *trilobatus* (yellow diamond). *Tometes siderocarajensis* (stars); orange star represents the type locality, black star the record before Tucuruí hydroelectric reservoir, and red stars the remaining localities. MCU: Mosaic of Conservation Units.

*Tometes siderocarajensis* is known to occur in the rapids of the Itacaiúnas River (Fig 7) and in its right-bank tributary, the Cateté River (average elevation of localities around 220 m a.s.l.), both located in the Mosaic of Conservation Units of Serra dos Carajás, Tocantins-Araguaia River Basin, State of Pará (Fig 8). In addition, *T*. *siderocarajensis* had its record confirmed in the Tocantins River based on specimens collected before the construction of the Tucuruí Hydroelectric Reservoir (INPA 52481), an area formerly known to contain many rapids but is currently flooded by the dam.

#### Remarks

*Tometes siderocarjensis* is typically found in rapids associated with rocky outcropping covered in aquatic macrophytes of the families Podostemaceae and Fabaceae, which act as a food source for these fish. The species is commonly caught by local, professional and amateur fishermen in the Itacaiúnas River. As bait, the fisherman use either leaves from Podostemaceae wrapped around a hood with sewing line, or hooks with the fruits of Fabaceae (Andrade, *pers*. *comm*.). Fishermen report that this fish provides a “good fight”, making it one of the most sought-after species for sport fishing in the region. Nevertheless, *T*. *siderocarjensis* is easily caught using gillnets placed around rapids [notice the vertical mark caused by gillnets on each specimen (Fig 4A, Fig 4B)].

One large specimen (GEA 1990, 340.0 mm SL) was dissected and the gastrointestinal contents examined. The stomach was completely full of undigested items, and free of parasites or aquatic macroinvertebrates. Large pieces of Podostemaceae (leaves and flowers) represented the vast majority of stomach contents, but we also found three small Fabaceae fruits (two of these cut in half without being crushed, and a third entire) and a cricket (Orthoptera) around 45 mm total length and split in half. The uncoiled intestine is long, and measures approximately four times the SL of the fish. Intestinal contents were composed of leaves (majority), flowers and seeds (a small amount), and abundance of Nematode fauna (mainly *Rondonia rondoni* Travassos 1919). It’s worth mentioning that the nematodes were only found in the last two thirds of the intestine, with a higher concentration in the second third.

The new species was only recorded in Tocantins-Araguaia drainages where it occurs syntopically with three other serrasalmid, rapids-adapted species, *Mylesinus paucisquamatus* Jégu and Santos 1988, *Myleus setiger* Müller and Troschel 1844, and *Tometes ancylorhynchus* Andrade, Jégu and Giarrizzo 2016. Whereas *M*. *setiger* and *T*. *ancylorhynchus* have soft and palatable meat, *T*. *siderocarajensis*, *T*. *kranponhah*, and *M*. *paucisquamatus* are locally referred as ‘pacu‒borracha’ (literally translated as ‘rubber pacu’), due to the rubbery texture of its flesh when cooked [11]. Notwithstanding, *T*. *siderocarajensis* is still consumed by fishermen of the Itacaiúnas River, due in part to its capacity to reach large sizes (~300 mm SL and up to 2 kg) relative to *M*. *paucisquamatus* (~ 200 mm SL and 400 g) and because it is an excellent food source that cannot be wasted. Furthermore, local human consumers dislike *M*. *paucisquamatus* because it tends to harbor higher abundances of endoparasitic fauna (Andrade, *pers*. *comm*.).

## Discussion

**Fig 9:**
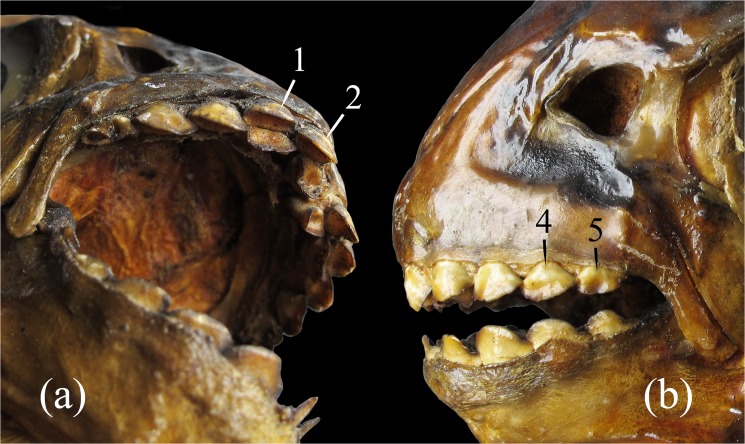
Teeth of *Tometes trilobatus*, (a) MNHN A.8650, lectotype, male, 337.3 mm SL, ventral view of premaxilla, (b) MNHN A.8649, paralectotype, female, 370.8 mm SL, lateral view. 1‒2: first and second teeth of labial premaxillary row; 4‒5: fourth and fifth teeth of lingual premaxillary row.

*Morphology* ‒ *Tometes siderocarajensis* has a darker body coloration, relative to its congeners with silvery gray coloration and noticeably silver-reddish overtones during the breeding period. Among its congeners, only large and live individuals of *T*. *lebaili* exhibit body coloration as dark as live specimens of *T*. *siderocarajensis*; however, Jégu et al. [32] figure 3 describes a large specimen of *T*. *lebaili* (~ 400 mm SL) presenting black coloration of pelvic fin with highlights of yellow. A similar coloration description for pelvic fins was noted in live specimens of *T*. *trilobatus* by Jégu et al. [12]: “*Toutes les nageoires sont noires*, *nettement plus foncées que le corps*” (all fins are black, evidently darker than the body color). However, *T*. *siderocarajensis* have distinctly dark pigmentation of pelvic fins in when compared to conspecifics (Fig 2A, Fig 2B). In addition, *T*. *trilobatus* and *T*. *lebaili* occur in left-bank tributaries of the Amazon Basin and in coastal drainages of the Guiana Shield, respectively (Fig 8), whereas *T*. *siderocarajensis* occurs exclusively in the Tocantins-Araguaia River Basin, within the Brazilian Shield drainage (Fig 8). *Tometes siderocarajensis* can be further distinguished from *T*. *trilobatus* and *T*. *lebaili* by having more circumpeduncular scale rows (39‒41 *vs*. 27‒34 and 32‒36, respectively), and further yet from *T*. *trilobatus* by the distinctive arrangement of its 1st and 2nd labial premaxillary teeth (Fig 3A and Fig 9A, respectively), and the strikingly different morphology of the 4th and 5th labial premaxillary teeth (Fig 3A and Fig 9B, respectively).

Besides the dark body coloration and densely pigmented with dark chromatophores pelvic fins, when compared to *T*. *ancylorhynchus*, which has distribution to Tocantins-Araguaia Basin, as well as *T*. *siderocarajensis* [formerly sympatric distribution, see under *Geographical distribution* and (Fig 8)], the new species can be differentiated by having more circumpeduncular scale rows (39‒41 *vs*. 30‒36, respectively). See more in key to *Tometes* species below.

When comparing *T*. *siderocarajensis* and two other sympatric serrasalmids of the *Myleus* clade (i.e. *Mylesinus paucisquamatus* and *Myleus setiger*), *T*. *siderocarajensis* is essentially distinguished from *M*. *paucisquamatus* by having thicker teeth that are strongly attached to the jaws (*vs*. very thin teeth, distinctly flattened anteroposteriorly, and weakly attached to the jaws), 4th and 5th teeth of the labial row that are clearly smaller than the 1st through 3rd teeth and with a sigmoid edge when viewed ventrally (*vs*. 4th and 5th teeth of the labial row equal in size as 1st through 3rd teeth, and all teeth of the labial row with aligned edges), and it also has more circumpeduncular scale rows (*vs*. 30‒34). While relative to *M*. *setiger*, *T*. *siderocarajensis* is strongly differentiated by the presence of a diastema between the two front teeth of the labial premaxillary row (*vs*. two front teeth making lateral contact or nearly so), and prepelvic serra formed by very thin and fragile spines pointing posteriorly from the belly (*vs*. a gradient of thicker, slightly curved spines that increase in size from anterior to posterior).

In the charge, *Tometes* species were largely confused with *Utiaritichthys* species [e.g. *T*. *ancylorhynchus* from Xingu and Toncantins-Araguaia River basins, *T*. *camunani* from Trombetas Basin and *T*. *kranponhah* from Xingu Basin (Fig 8)]. The paratypes of *T*. *siderocarajensis* cataloged under MZUSP 117052, collected by Michael Goulding in the early 1980s at Itacaiúnas River, were misidentified as *Utiaritichthys sennaebragai*. Although *U*. *sennaebragai* is recognized only to Tapajós River Basin and occurrences outside of this basin are considered to be misidentifications [11,33], species are still reported in other watersheds such as Xingu, Tocantins-Araguaia, Madeira, Orinoco, and others [34]. This was most likely influenced by Gosline [13], which diagnosed a serrasalmid specimen (catalog CAS 20222) with a poorly-developed abdominal serra as *U*. *sennaebragai*. This diagnosis was recently changed to *T*. *ancylorhynchus* by Andrade et al. [11]. It is noteworthy that this lot as well as INPA 52481 (*T*. *siderocarajensis*), came from an area where the rapids of the Tocantins-Araguaia River Basin were formerly located, at the cities of Marabá and Itupiranga, respectively. However, most of this area is currently submersed by the Tucuruí reservoir (Fig 8). Due to the loss of rapid stretches and flooded areas on the lower Tocantins River, the possibility of finding rheophilic fish is remote.

*Molecular analysis* ‒ The isolated application of a single technique for description of a novel species (i.e. morphological or genetic analysis alone), has been criticized and contains several caveats, when a small number of individuals per species are used or only a small fraction of the global richness is considered [35]. The present study is the first to use DNA barcode methodology to assist in the description of a new species of Serrasalmidae. Despite the low number of samples per species, the DNA barcoding analysis of 28 specimens representing the entire *Tometes* genus was effective, and allowed for the correct discrimination of all analyzed species.

The mean of intra- and interspecific distances were 0.2% and 5.8%, respectively, and differed from studies of Pereira et al. [27] and Castro Paz et al. [36] for freshwater fishes in South America, which found averages of 1.3% and 6.8% and 2.3% and 19.3%, respectively. Thus, the mean interspecific distance found among *Tometes* species is low (5.8%) compared to the global average found in studies of freshwater fishes in North America and in the Neotropical region (6.8%). The average of the intraspecific distances was even lower than those found for fishes of the Neotropics and other regions [27,37–39]. This relatively low interspecific distance may reflect a recent divergence experienced by *Tometes* species. A similar result was obtained by Toffoli et al. [40] for freshwater stingrays in the Amazon Basin. However, the *Tometes* species are distinguished by their morphology. Except for *T*. *makue* and *T*. *lebaili*, all other *Tometes* species have low interspecific distances (mean 3.3%). Montoya-Burgos et al. [41] working with *Hypostomus* and Hubert et al. [42] studying two serrasalmid genera representative of the piranhas (i.e. *Serrasalmus* spp. and *Pygocentrus* spp.), proposed a hypothesis of radiation of these groups, which may have originated from 2 to 12 MYA. These authors also suggest that the low distance pattern can be found in other Neotropical fish groups, and is indicative of recent diversification. Because approximately 70% of the comparisons among *Tometes* species showed less divergence than 6% (S1 Table), our results were consistent with this pattern. So, *T*. *makue* from Negro River and *T*. *lebaili* from Maroni River were recovered as sister species, whereas *T*. *ancylorhynchus* from Araguaia was recovered as the close taxa close to *T*. *kranponhah* from Xingu (Fig 5). The interspecific molecular distance between *T*. *trilobatus* from Jari River (left-bank tributary of Amazon River Basin) and the *T*. *siderocarajensis* from Itacaiúnas River (sub-basin of lower Tocantins-Araguaia River Basin) reveals the recent divergence between these taxa, and agrees with hypothesis of diversification of freshwater species [43]. *Tometes*, as well as the genera *Mylesinus*, *Ossubtus* and *Myleus* (*stricto sensu* [44]), are highly rheophilic serrasalmid fishes with high degrees of endemism since most of their representative species are restricted to a few or even a single river basin.

Although the divergence between *T*. *trilobatus* and *T*. *siderocarajensis* is below the barcoding threshold (2%) for interspecific distinction, all species showed particular diagnostic nucleotides. *Tometes siderocarajensis* had two diagnostic nucleotides in 83 informative sites for the COI gene, and the same was observed to *T*. *trilobatus*, which was distinguished by also having two diagnostic nucleotides distinctive from the new species. According to Birstein et al. [45], using diagnostic nucleotides for comparisons of closely related species is more difficult, since the nucleotide composition is more similar. *Tometes siderocarajensis* presented two nucleotide sequences as diagnostic sites for the COI gene, sites 594 (T/A) and 696 (A/G), and *T*. *trilobatus* presented the diagnostic sites 120 (A/G) and 180 (G/A), which corroborate with the proposal of a new serrasalmid taxa (S2 Table). Therefore, *Tometes siderocarajensis* should be considered a distinct species within the *Tometes* genus due to both morphological and molecular characteristics that distinguish it from congeners. The data suggest that the new species is monophyletic, and clearly diagnosable from other species of *Tometes* by morphological and molecular autapomorphies. This leads us to conclude that *T*. *siderocarajensis* is following a unique evolutionary trajectory under the phylogenetic species concept [28], and thus merits the status of a valid novel species.

*Distribution pattern and conservation* ‒ Despite the fact that other serrasalmids *M*. *paucisquamatus*, *M*. *setiger* and its congener *T*. *ancylorhynchus* occur in the same Tocantins-Araguaia River Basin, *T*. *siderocarajensis* is the only *Tometes* species currently known to occur in Itacaiúnas sub-basin. The species *T*. *ancylorhynchus* (INPA 3134) and *T*. *siderocarajensis* (INPA 52481) were last documented to co-occur in the lower Tocatins River (Fig 8) in 1980, but have not been found there since this area was flooded by the hydroelectric dam. *Tometes ancylorhynchus*, which also occurs in the Xingu and Tocantins-Araguaia River basins [11], apparently does not occur in the sub-basin of the Itacaiúnas River. On the other hand, *M*. *paucisquamatus*, which is endemic of the Tocantins-Araguaia, is widespread throughout this basin, since it is found along with the two aforementioned species. Although possibility a result of habitat loss, *T*. *siderocarajensis* is supposedly endemic to the Itacaiúnas sub-basin, which is the main tributary of the Tocantins River that drains the Carajás mineral province [46]. Taking into account that the Tocantins-Araguaia Basin is strongly modified by hydroelectric dams, the Itacaiúnas sub-basin has been constantly degraded by the effects of mining, soybean-farming and cattle ranching [46], and that the distribution of *T*. *siderocarajensis* is restricted to the rapids of this sub-basin, we stress the importance of the Mosaic of Conservation Units of the Serra dos Carajás as a protected area for whole biodiversity of the Itacaiúnas sub-basin. We continue to recommend the protection of rapids of the Serra dos Carajás to ensure the presence of rheophilic species.

### Key to species of *Tometes* Valenciennes 1850

1 Mouth terminal to slight subinferior … 2
1’ Mouth clearly oblique upward turned … *T*. *lebaili* (Atlantic coastal drainages occurring in French Guiana rivers of Litany, Mana, Maroni, and Tampoc, as well as in Suriname rivers of Commewine, Oulemary, and Tapanahony)
2 First and second teeth of the premaxillary labial row with evident lateral contact … 3
2’ First and second teeth of the premaxillary labial row clearly laterally spaced … 4
3 Twenty-seven to 34 circumpeduncular scales, first and second premaxillary labial teeth lacking lateral cusps, no defined marks on opercle … *T*. *trilobatus* (Oiapoque River drainage between French Guiana and Brazil, and in the Northeastern Brazilian Rivers of Anotaié, Araguari, Cassiporé, Flexal, and Jari)
3’ Thirty-eight to 43 circumpeduncular scales, lateral cusps on first and second premaxillary labial teeth, tear-drop black blotch on opercle … *T*. *kranponhah* (Xingu River Basin, such as drainages of the Iriri, Xingu, Bacajá rivers)
4 Prepelvic spines counting from 11 to more … 5
4’ Prepelvic spines ranging from none to 9 … *T*. *makue* (Negro River Basin in Brazil, and Orinoco River Basin in Venezuela)
5 Circumpeduncular scale rows counting from 37 to more … 6
5’ Circumpeduncular scale rows ranging from 30 to 36 … *T*. *ancylorhynchus* (Tributaries of the Xingu River Basin and Tocantins-Araguaia River Basin, except Itacaiúnas River drainage)
6 Pelvic fins hyaline, ground body coloration predominantly silver, 10 to 11 predorsal vertebrae, and six to eight supraneurals … *T*. *camunani* (upper Trombetas River Basin)
6’ Pelvic fins black pigmented from the middle of fin to its tip or totally black pigmented, ground body coloration predominantly blackened, nine predorsal vertebrae, and five supraneurals … *T*. *siderocarajensis* (Rio Tocantins-Araguaia Basin at Itacaiúnas River and former occurrence in Tocantins River at area flooded by Tucuruí reservoir)

## Supporting information

S1 Table Minimum *p*-distances between *Tometes* species. Molecular distances are based on the 580-bp fragment of mtDNA COI.
(DOCX)

S2 Table Species level diagnostic characters observed in the mtDNA COI gene of *Tometes siderocarajensis* and its congeners. First line indicates position of the character within the mtDNA COI gene.
(DOCX)

S1 Comparative material examined (DOCX)
