# Supplementary material for: A new species of Tometes Valenciennes 1850 (Characiformes: Serrasalmidae) from Tocantins-Araguaia River Basin based on integrative analysis of molecular and morphological data
Source: PLoS One. 2017 Apr 19;12(4):e0170053. doi: 10.1371/journal.pone.0170053 (PMC5396854; doi:10.1371/journal.pone.0170053)
Supplement: S1 Table — Molecular distances are based on the 580-bp fragment of mtDNA COI. (DOCX) [file pone.0170053.s001.docx]

**Table S1. Minimum p-distances between *Tometes* species. Molecular distances are based on the 580-bp fragment of mtDNA COI.**

| mtDNA COI | 1 | 2 | 3 | 4 | 5 | 6 |
| --- | --- | --- | --- | --- | --- | --- |
| 1. *T. siderocarajensis* **sp. nov.** |  |  |  |  |  |  |
| 2. *T. ancylorhynchus* | 0.056 |  |  |  |  |  |
| 3. *T. kranponhah* | 0.027 | 0.027 |  |  |  |  |
| 4. *T. camunani* | 0.031 | 0.053 | 0.037 |  |  |  |
| 5. *T. lebaili* | 0.073 | 0.073 | 0.067 | 0.068 |  |  |
| 6. *T. makue* | 0.091 | 0.093 | 0.079 | 0.074 | 0.080 |  |
| 7. *T. trilobatus* | 0.016 | 0.052 | 0.034 | 0.037 | 0.079 | 0.089 |
