# Supplementary material for: A new species of Tometes Valenciennes 1850 (Characiformes: Serrasalmidae) from Tocantins-Araguaia River Basin based on integrative analysis of molecular and morphological data
Source: PLoS One. 2017 Apr 19;12(4):e0170053. doi: 10.1371/journal.pone.0170053 (PMC5396854; doi:10.1371/journal.pone.0170053)
Supplement: S2 Table — First line indicates position of the character within the mtDNA COI gene. (DOCX) [file pone.0170053.s002.docx]

**Table S2. Species level diagnostic characters observed in the mtDNA COI gene of *Tometes siderocarajensis* sp. nov. and its congeners. First line indicates position of the character within the mtDNA COI gene.**

|  | 120 | 180 | 594 | 696 |
| --- | --- | --- | --- | --- |
| 1. *Tometes siderocarajensis* sp*.* nov*.* | G | A | T | A |
| 2. *Tometes ancylorhynchus* | G | A | A | G |
| 3. *Tometes kranponhah* | G | A | A | G |
| 4. *Tometes camunani* | G | A | A | G |
| 5. *Tometes lebaili* | G | A | A | G |
| 6. *Tometes makue* | G | A | G | G |
| 7. *Tometes trilobatus* | A | G | A | G |
