# Supplementary material for: A new species of Tometes Valenciennes 1850 (Characiformes: Serrasalmidae) from Tocantins-Araguaia River Basin based on integrative analysis of molecular and morphological data
Source: PLoS One. 2017 Apr 19;12(4):e0170053. doi: 10.1371/journal.pone.0170053 (PMC5396854; doi:10.1371/journal.pone.0170053)
Supplement: S1 Comparative material examined — (DOCX) [file pone.0170053.s003.docx]

**Comparative material examined*.***

*Mylesinus paucisquamatus*: All from Brazil, Pará, GEA 836 (1, 159.4 mm SL), Parauapebas, rio Itacaiúnas. INPA 1808 (holotype, 162 mm SL), Tucuruí, rio Tocantins, Jatobal. MNHN 1988-1705 (2 paratypes, 131.7‒137.9 mm SL); MNHN 1988-1706 (4 paratypes, 112.8‒185.1 mm SL); MNHN 1988-1707 (1 paratype, 165.3 mm SL), Tucuruí, rio Tocantins, Jatobal. MZUSP 83996 (5, 38.3‒102.1 mm SL), Novo Jardim, Lagoa bonita, marginal lake of rio Palmeiras.

*Myleus setiger*: BMNH 1971.5.10.65 (1 syntype, 182.9 mm SL), British Guiana. GEA 1963 (1, 176.3 mm SL), Brazil, Pará, Altamira, Xingu Basin, rio Bacajá. GEA 1971 (1 skel., 137.5 mm SL), Brazil, rio Xingu. GEA 1972 (1 head, skel., 45 mm HL), Brazil, Ourilândia do Norte, Itacaiúnas Basin, rio Cateté. MNHN A.8629 (1 stuffed specimen, holotype of *Myletes divaricatus*, 186.7 mm SL), Guyana, Essequibo River. MNHN A.9868 (, holotype of *Myletes doidyxodon* 137.9 mm SL), Brazil, Amazon River. MZUSP 40489 (1, 87.4 mm SL), Goiás, Flores de Goiás, rio Paranã (cachoeira), Fazenda Olho D'água.

*Tometes ancylorhynchus*: All from Brazil. CAS 20222 (1, 162 mm SL), Marabá, rio Tocantins. GEA 1714 (1 skel., 126 mm SL), Vitória do Xingu, rio Xingu, Cachoeira do Jericoá. GEA 1949 (1 paratype, 148.7 mm SL), Altamira, rio Iriri, rapids downstream of Cachoeira Grande. GEA 1955 (2, 156‒158.5 mm SL), Altamira, rio Xingu, rapids of Kaituká. INPA 2356 (1, 187.8 mm SL), Tucuruí, rio Tocantins, upstream of Tucuruí Hydroelectric reservoir. INPA 3633 (15, 86.3–193.9 mm SL); INPA 3634 (3, 169.1–208.5 mm SL) Itupiranga, rio Tocantins, upstream of Tucuruí Hydreletric reservoir. INPA 4485 (1, 141.5 mm SL); INPA 4505 (1, 177.0 mm SL), Tucuruí, rio Tocantins, Tucuruí Hydroelectric reservoir; INPA 5134 (3, 124.1–162.4 mm SL), Itupiranga, rio Tocantins; INPA 52482 (6, 147.7–175.3 mm SL), Araguatins, rio Araguaia, Santa Isabel Hydroelectric reservoir. MNHN 1999–1168 (2 paratypes, 101.9–174.8 mm SL), rio Xingu. MPEG 31014 (holotype, 150.4 mm SL), Vitória do Xingu, rio Xingu, Cachoeira do Jericoá. MPEG 31015 (7 paratypes, 91.2–152.5 mm SL), Altamira, rio Xingu, Pedral do Roboque Velho. MPEG 31016 (1 paratype, 159.7 mm SL), Altamira, rio Xingu, Robojinho, Cachoeira do Porfírio. ZUEC 10023 (1 paratype, 118.7 mm SL), Altamira, rio Xingu.

*Tometes camunani*: All from Brazil, Pará, MPEG 23447 (holotype, 224.3 mm SL), rio Erepecuru. MPEG 23439 (1 paratype, 248.0 mm SL); MPEG 23440 (1 paratype, 384.0 mm SL); MPEG 23441 (1 paratype, 276.0 mm SL); MPEG 23443 (2 paratypes, 247.0‒293.0 mm SL); MPEG 23444 (3 paratypes, 291.0‒329.0 mm SL), rio Trombetas. MPEG 23448 (1 skel., 382.0 mm SL), rio Trombetas. INPA 3639 (1 paratype, 299.0 mm SL), rio Trombetas.

*Tometes kranponhah*: All from Brazil, Pará, Altamira, Xingu basin. ANSP 193019 (1 paratype, 153.8 mm SL); ANSP 193019 (1 paratype, 345.0 mm SL), rio Iriri, Cachoeira Grande do Iriri. ANSP 194659 (1 paratype, 250.0 mm SL), rio Iriri, below Cachoeira Grande do Iriri. GEA 1939 (1 skel., 305.0 mm SL), rio Xingu, Pedral do Roboque Velho. MPEG 31000 (holotype, 132.3 mm SL), rio Xingu, Cachoeira do Espelho. MPEG 31001 (5 paratypes, 257.5–319.0 mm SL), rio Xingu, Pedral do Roboque Velho. MPEG 31003 (3 paratypes, 304.6–350.0 mm SL); MPEG 31004 (1 paratype, 234.9 mm SL); MPEG 31006 (1 paratype, 320.1 mm SL), rio Bacajá. MZUSP 105645 (1 paratype, 207.8 mm SL), rio Xingu, Cnec. MZUSP 110948 (1 skel., 267.0 mm SL), rio Iriri, Cachoeira Grande.

*Tometes lebaili*: IRSNB 21.343 (1, 400.5 mm SL), Suriname, Marowijne, Paloemeu River, Papadronsoela. MNHN 1993-3452 (1, 251.0 mm SL), French Guyana, St. Laurent du Maroni, Maripasoula, Maroni River. MNHN 2000-6038 (2 paratypes, 128.5‒146.1 mm SL); MNHN 2001-1212 (1 paratype, 147.4 mm SL); MNHN 2001-1213 (1, 213.1 mm SL); MNHN 2001-1215 (1 paratype, 219.4 mm SL); MNHN 2001-2384 (holotype, 106.7 mm SL), French Guiana, Maroni River.

*Tometes makue*: All from Brazil, Amazonas, São Gabriel da Cachoeira, rio Negro. INPA 7344 (holotype, 240.0 mm SL); INPA 3179 (1, 125.0 mm SL); INPA 4913 (1, 370.0 mm SL); INPA 4914 (2, 330.0‒355.0 mm SL); INPA 4915 (2, 138.0‒158.0 mm SL); INPA 4916 (2, 239.0‒307.0 mm SL); INPA 4917 (1, 261.0 mm SL); INPA 4920 (4, 172.0‒229.0 mm SL); MNHN 2001-2712 (3 paratypes, 208.2‒230.9 mm SL). INPA 43077 (1, 57.2 mm SL).

*Tometes trilobatus*: MNHN A.8649 (1 stuffed specimen, Paralectotype, 370.0 mm SL); MNHN A.8650 (1 stuffed specimen, Lectotype, 337.3 mm SL); MNHN A.8651 (1 stuffed specimen, holotype of *Tometes unilobatus*, 277.0 mm SL), French Guiana, Cayenne. MNHN 1998-0099 (1, 248.0 mm SL), French Guyana, Cayenne, Saut Maripa, Oyapock river. IEPA 2853 (1, 277.9 mm SL), Brazil, Amapá, Rio Anotaié, Oiapoque. IEPA 3564 (1, 85.4 mm SL); IEPA 3568 (1, 107.5 mm SL); IEPA 3570 (1, 89.8 mm SL), Brazil, Amapá, rio Araguari, UHE de Ferreira Gomes. IEPA 3577 (1, 422.0 mm SL), Brazil, Amapá, Rio Cassiporé. IEPA 4303 (1, 263.1 mm SL), Brazil, Amapá, rio Jari, Laranjal do Jari. INPA 19967 (1, 116.0 mm SL), Brazil, Amapá, rio Araguari, Cachoeira Santa Rosa. ZMA 107.687 (1, 108.8 mm SL), French Guiana and Brazil, Oyapock Basin, near of confluence with Crique Armontabo.
